# OrthoDB and BUSCO update: annotation of orthologs with wider sampling of genomes

**DOI:** 10.1093/nar/gkae987

**Published:** 2024-11-13

**Authors:** Fredrik Tegenfeldt, Dmitry Kuznetsov, Mosè Manni, Matthew Berkeley, Evgeny M Zdobnov, Evgenia V Kriventseva

**Affiliations:** Department of Genetic Medicine and Development, University of Geneva Medical School, rue Michel-Servet 1, 1211 Geneva, Switzerland, and Swiss Institute of Bioinformatics, rue Michel-Servet 1, 1211 Geneva, Switzerland; Department of Genetic Medicine and Development, University of Geneva Medical School, rue Michel-Servet 1, 1211 Geneva, Switzerland, and Swiss Institute of Bioinformatics, rue Michel-Servet 1, 1211 Geneva, Switzerland; Department of Genetic Medicine and Development, University of Geneva Medical School, rue Michel-Servet 1, 1211 Geneva, Switzerland, and Swiss Institute of Bioinformatics, rue Michel-Servet 1, 1211 Geneva, Switzerland; Department of Genetic Medicine and Development, University of Geneva Medical School, rue Michel-Servet 1, 1211 Geneva, Switzerland, and Swiss Institute of Bioinformatics, rue Michel-Servet 1, 1211 Geneva, Switzerland; Department of Genetic Medicine and Development, University of Geneva Medical School, rue Michel-Servet 1, 1211 Geneva, Switzerland, and Swiss Institute of Bioinformatics, rue Michel-Servet 1, 1211 Geneva, Switzerland; Department of Genetic Medicine and Development, University of Geneva Medical School, rue Michel-Servet 1, 1211 Geneva, Switzerland, and Swiss Institute of Bioinformatics, rue Michel-Servet 1, 1211 Geneva, Switzerland

## Abstract

OrthoDB (https://www.orthodb.org) offers evolutionary and functional annotations of orthologous genes in the widest sampling of eukaryotes, prokaryotes, and viruses, extending experimental gene function knowledge to newly sequenced genomes. We collect gene annotations, delineate hierarchical gene orthology and annotate the orthologous groups (OGs) with functional and evolutionary traits. OrthoDB is the leading resource for species diversity, striving to sample the most diverse and well-researched organisms with the highest quality genomic data. This update expands to include 5827 eukaryotic genomes. We have also added coding DNA sequences (CDSs) and gene loci coordinates. OrthoDB can be browsed, downloaded, or accessed using REST API, SPARQL/RDF and now also via API packages for Python and R Bioconductor. OrthoLoger (https://orthologer.ezlab.org), the tool used for inferring orthologs in OrthoDB, is now available as a Conda package and through BioContainers. ODB-mapper, a component of OrthoLoger, streamlines annotation of genes from newly sequenced genomes with OrthoDB evolutionary and functional descriptors. The benchmarking sets of universal single-copy orthologs (BUSCO), derived from OrthoDB, had correspondingly a major update. The BUSCO tool (https://busco.ezlab.org) has become a standard in genomics, uniquely capable of assessing both eukaryotic and prokaryotic species. It is applicable to gene sets, transcriptomes, genome assemblies and metagenomic bins.

## Graphical Abstract

**Graphical Abstract:**
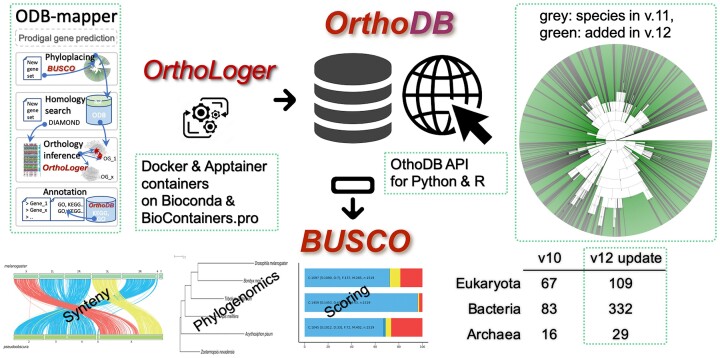

## Introduction

Genomics continues to uncover the vast space of genomic sequences, underlying life's diversity. As the volume of genome data expands at an unprecedented rate, the challenge of interpreting this information becomes increasingly complex. Gene orthology emerged as a crucial concept in navigating this expanding landscape. Orthologs — genes in different species originating from a gene of their last common ancestor — mostly retain their ancestral functions across different species, providing a framework for comparative genomics and functional prediction (1,2). The identification of orthologous genes serves as a cornerstone for numerous applications in genomics, from evolutionary studies to functional annotation of newly sequenced genomes. It allows hypothesizing about gene functions in less-studied organisms based on knowledge from well-characterized model species. The relevance of this approach continues to grow as the rate of sequence generation far outpaces our ability to experimentally characterize each gene. However, the identification of orthologs is intricate, leading to a variety of methods with varying trade-offs in precision, sensitivity, and scalability, as well as databases providing precomputed orthology data (3–8).

Gene homology, defined as the evolutionary relatedness of genes, commonly serves as a basis for hypothesizing functional similarities among genes inherited from their ancestors. This principle underpins the use of clusters of homologous genes in databases like Pfam (9). Orthology refines this concept by anchoring homology to the last common ancestor (LCA), grouping genes that presumably originated from a specific phylogenetic radiation. Thus, each gene from the LCA initiates an orthologous group (OG), which includes all descendant genes across different species that evolved from that ancestral gene. This refinement effectively narrows the functional scope compared to broader families of homologs, which also include all genes that arose from gene duplications before the LCA. Therefore, orthologous groups provide more specific functional inferences than broader families of homologs. The concept is hierarchical (2,10–12). Inferring OGs at different taxonomic levels corresponding to distinct LCAs enables the identification of finer-grained OGs in more recent species radiations and broader groups for more ancestral LCAs, leading to more precise or more generalized functional inferences, respectively. Best reciprocally matching genes are often used as an approximation of pairwise orthology, despite overlooking duplications that occur after the speciation of the pair. It's important to note that OGs at a specific taxonomic level are not merely a collection of pairwise orthologs between species within that clade. Some pairs may have diverged more recently than the taxonomic level of the OGs. Therefore, pairwise inferences, referring to different LCAs, cannot be directly compared with OGs that reference a common, more ancestral LCA of the clade. Evolutionary histories of multigene families of homologs can be complex, and striving for more specificity generally results in more over-splitting of OGs. In contrast, approaches like OrthoMCL (13) and OrthoFinder (14) favor inclusivity, increasing sensitivity but reducing specificity, leading to results that encompass broader families of homologs. OrthoDB (https://www.orthodb.org) is balancing sensitivity and specificity to optimize accuracy of functional inferences. OrthoDB provides extensive genomic diversity coverage with computed gene ortholog evolutionary traits and functional annotations to support comparative genomic studies. Key features include interactive comparative charts, the ability to map user data, a user-friendly web interface, and access through REST API and SPARQL RDF, making OrthoDB a useful resource for genomic research.

### OrthoDB update

**Figure 1.**
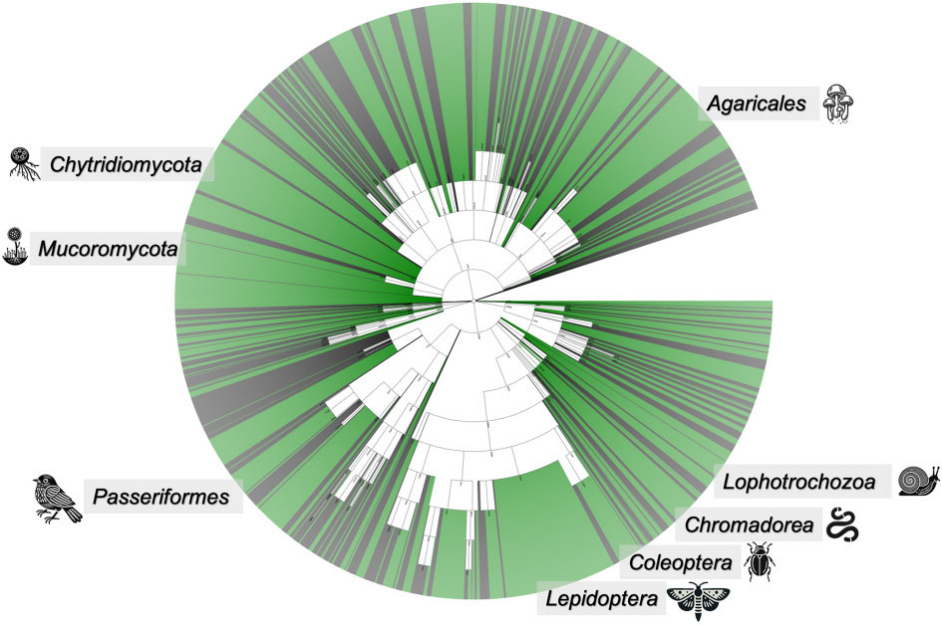
The expansion of OrthoDB’s Eukaryota taxonomic coverage is visualized using Newick Utilities (27). Grey-colored branches represent species sampled in version 11, while green branches indicate those added in version 12. The internal nodes correspond to the levels of orthology sampled from the NCBI taxonomy. Labeled in the figure are the most notable expansions, including Lepidoptera moths, Chromadorea roundworms, Chytridiomycota fungi with flagellated spores, Mucoromycota molds, Passeriformes songbirds, Coleoptera beetles, Lophotrochozoa invertebrates and Agaricales mushrooms, among others.

This update to OrthoDB version 12 significantly expands its coverage of Eukaryotes, nearly tripling the count to 5827 genomes (Figure 1). This pushed the overall number of annotated genes to increase from 100 to 162 millions. Comprehensive genomic diversity coverage is crucial for the effectiveness of any orthology resource. Incorporating a wide spectrum of evolutionary lineages, facilitates more accurate and reliable ortholog identification as it enhances both the accuracy and reliability of the deductions made. More diverse genomic datasets also enable more comprehensive and precise functional annotations, which are the focal points for predictive models and comparative genomics. However, striving for extensive coverage increases the demands on computational resources and challenges the scalability of the underlying orthology delineation tools. Table 1 records the recent developments in genomic sampling by orthology resources with the broadest coverage (4,6–8). The most significant expansions in this OrthoDB update include Lepidoptera moths (10×), Chromadorea roundworms (9×), Chytridiomycota fungi with flagellated spores (8×), Mucoromycota molds (7×), Passeriformes songbirds (6×), Coleoptera beetles (5×), Lophotrochozoa invertebrates (5×), Araneae spiders (5×) and Phytophthora water molds (5×), Agaricales mushrooms (5×), among others.

**Table 1.** Phylogenetic coverage growth

	OrthoDB.v12	OMA	OrthoDB.v11	eggNOG.v6	KEGG-OC	OrthoDB.v10	eggNOG.v5
Release	2024	2024	2022	2022	2019	2018	2018
Eukaryota	5827	713	1952	1322	456	1271	477
Bacteria	17 551	1965	17 551	10 756	4880	5609	4445
Archaea	607	173	607	457	278	404	168

We selected the most diverse organisms with the highest quality of genomic data and the most extensive functional gene annotations, reflecting on the rapid expansion of the genomics field. Our genome sampling procedure identifies well-sampled taxonomic clusters that exhibit high pairwise genomic identity using MASH (8). For each of these clusters, we select a representative genome that is both the most annotated and the most complete in terms of BUSCO (15). We relied on OrthoLoger software (16) for *de novo* delineation of orthologous genes at various levels of orthology, according to NCBI Taxonomy (17). An additional 1740 complete genomes, which did not pass the initial selection, have then been mapped to these newly defined orthologous groups. These are marked with an ‘M’ on the interactive OrthoDB taxonomy tree in the ‘Advanced’ section of the OrthoDB web user interface. This taxonomy tree lists all species available in OrthoDB, together with the genome assembly used. Genome assembly accession numbers have been made searchable in this update, in addition to the species names, in the search field above the taxonomy tree. Protein-coding gene sequences were retrieved from the union of complete genomes in RefSeq v220 as of 20 September 2023 (18), GenBank v257 as of 25 August 2023 (19), and Ensembl Rapid Release as of 15 October 2023 (20).

Orthologous groups (OGs) presumably represent genes of the last common ancestor at each taxonomic level. These OGs are annotated with evolutionary and functional descriptors, based on the foundational model of gene function conservation. The functional descriptors for each OG are aggregated from individual gene annotation records, including annotations and cross-references from UniProt (21), and aim to concisely and precisely outline functional knowledge in human-readable language. The summary statistics of Gene Ontology terms (22), InterPro protein domains (23) and, whenever possible, COG functional categories (5), KEGG pathways (24) and enzyme EC numbers provides further insight into likely functions of the OG genes. Such high-level functional descriptors are helpful for comparative studies and metagenomics. While the collated functional gene annotations provide a wealth of information, they may contain inaccuracies. Aggregating data per OG can highlight errors in the underlying data; therefore, discordant annotations should be approached with caution. Evolutionary descriptions for each OG were derived from genomic data and gene sequence alignment metrics. These include: (i) the ‘phyletic profile’, which indicates gene universality (the proportion of species with orthologs) and duplicability (the proportion of multi-copy versus single-copy orthologs), (ii) the ‘evolutionary rate’, which reflects the relative degree of protein sequence conservation or divergence and (iii) ‘sibling groups’. The universality of a gene family suggests a broadly necessary functional role, whereas lineage-restricted genes may be driven by lineage-specific adaptations. Duplicability often correlates with molecular function types, such as components of a signal-transduction pathway or a protein complex, which may be under single-copy control (25). These OrthoDB unique annotations provide a valuable evolutionary perspective. In this release, we added coding DNA sequences (CDSs) to the database, in addition to amino acid sequences, except for polyprotein viruses. These are available via the ‘View CDS fasta’ link in OG headers and in ‘gene view’, which is accessible by querying gene identifiers with the ‘get gene’ search selector. We also included gene genomic location coordinates. These loci coordinates facilitate navigation to gene contexts in genome browsers and enable the exploration of conserved ortholog arrangements using synteny viewers. Although the visualization of conserved synteny blocks, introduced in an earlier version of OrthoDB (26), saw limited use resulting in discontinued support, the availability of loci coordinate data through the API will support renewed interest in exploring conserved blocks of ortholog arrangements.

The OrthoDB web interface offers three views: (i) a list of orthologous groups related to a user query, (ii) a detailed view of an OG that includes a Sankey diagram to simplify navigation through the hierarchy of orthology, functional and evolutionary descriptors of the OG, and a list of member orthologs within an interactive organism taxonomy structure and (iii) a gene-centric view showing pairwise orthology between the query gene and other sampled species. Functional descriptions and cross-references for each gene, which are hidden by default, can be revealed by clicking ‘>>’, with the size of the chevrons indicating the volume of available annotations. The search functionality supports autocomplete for single keywords and phrases and allows for complex queries combining multiple keywords with logical operations (e.g. ‘-’ or ‘!’ for logical NOT). To search for an exact phrase, double quotation marks should be used (e.g. ‘Cytochrome P450’). The ‘Advanced’ panel enables users to customize the default expanded species and filter by organismal taxonomy and orthology level, selecting appropriate nodes on the searchable species tree. Users can also exclude certain clades by adding a negated taxonomic node name to the search field (e.g. ‘kinase !Metazoa’ with the Eukaryota level selected targets specific kinases absent in Metazoa). For repeated queries, users can save complex filter setups via a link labeled ‘Bookmark OrthoDB’ located in the top-right corner. This link, a JavaScript bookmarklet, allows quick searches on OrthoDB using saved settings. Additionally, users can highlight a keyword on any web page and click the saved bookmarklet to initiate a search for that keyword in OrthoDB.

To facilitate programmatic access to OrthoDB data, in addition to the REST web API, we have developed a Python wrapper, OrthoDB-py available from https://gitlab.com/ezlab/orthodb_py, and an R wrapper OrthoDB-R available from https://gitlab.com/ezlab/orthodb_r, soon to be available as a Bioconductor package. Python and R are among the most widely used languages in bioinformatics. Providing these software packages ensures that a broader research community can utilize OrthoDB within their preferred programming environments, abstracting the complexities of direct web API interactions.

### OrthoLoger update

Orthology delineation is crucial for comparative studies of newly sequenced genomes. For this purpose, we developed the OrthoLoger software. Benchmarking, as previously described (7), showed that OrthoLoger, OrthoFinder (14) and SonicParanoid (28) perform similarly, with OrthoFinder slightly favoring sensitivity and SonicParanoid slightly favoring specificity.

OrthoLoger offers *ab initio* ortholog prediction and a hierarchical mode guided by a user-provided species tree/taxonomy that improves scalability and consistency across different levels of orthology. This hierarchical mode was used for the OrthoDB v12 update.

**Figure 2.**
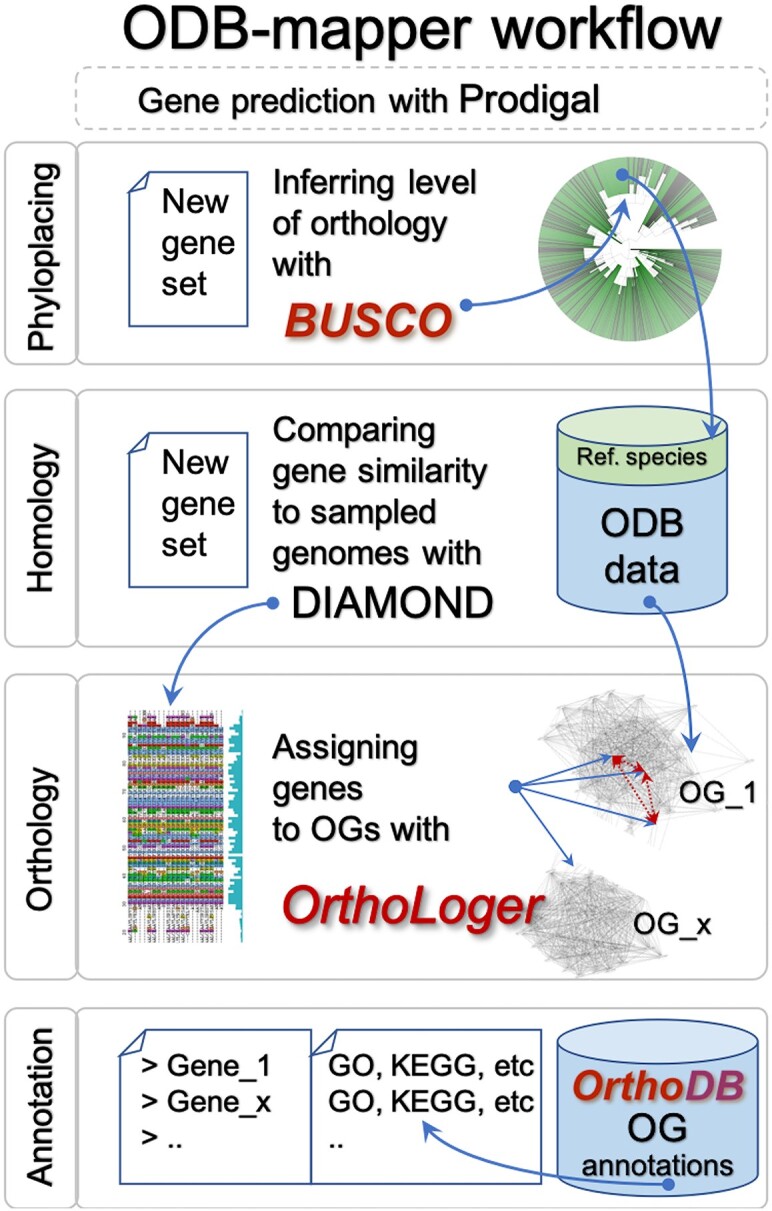
Visualized workflow of the ODB-mapper, which leverages OG annotations for streamlined, tentative characterization of newly sequenced genomes. The process begins with phyloplacing of user data to narrow down the taxonomic range, followed by homology searches within this range. Subsequently, new genes are mapped to OrthoDB-delineated OGs and are then annotated with the OGs' functional and evolutionary descriptors.

With the ODB-mapper script (Figure 2) OrthoLoger provides a streamlined approach for annotating genes in newly sequenced genomes, which assigns genes to pre-computed and annotated orthologous groups in OrthoDB. This method is also effective for lower-quality input data, such as incomplete gene sampling from transcriptomes, helping to minimize errors. This method is also effective for lower-quality input data, such as incomplete gene sampling from transcriptomes, helping to minimize errors. When provided with microbial-sized DNA sequence inputs, ODB-mapper first attempts gene prediction using Prodigal (29), making it directly applicable to the functional annotation of metagenome assembled genomes (MAGs). OrthoLoger is freely available at https://orthologer.ezlab.org. For enhanced usability, OrthoLoger is now available as both Docker and Apptainer containers, through a Bioconda channel, and from the BioContainers.pro registry (30) available from https://biocontainers.pro/tools/orthologer. Containers encapsulate software with all its dependencies, making it platform-independent and ensures that the tool can be run consistently across different computing environments. Providing OrthoLoger as a Conda package allows for easy integration into bioinformatics workflows. Conda is a popular package management system in the bioinformatics community, known for handling complex dependency trees and simplifying software management. Listing OrthoLoger on BioContainers.pro aligns it with a standardized registry, enhancing its visibility and accessibility to the scientific community. This registry is widely used for discovering and deploying bioinformatics tools, ensuring that OrthoLoger reaches a broader audience.

### BUSCO update

BUSCO (Benchmarking Universal Single-Copy Orthologs) has become a standard in genomics for quality control and comparative analyses (15). The method is based on the evolutionary concept that all species are expected to possess certain highly conserved core genes (25). Focusing on the fraction of genes typically present in single copy allows also to estimate artifactual genome assembly duplications.

Since 2012, OrthoDB has provided curated BUSCO datasets of single-copy orthologs expected to be present across specific taxonomic lineages (e.g. vertebrates, insects, fungi) (26). The BUSCO software searches for these genes in the provided genome, gene set or transcriptome, classifying them as complete, fragmented, or missing, and identifying potential duplications. The resulting BUSCO score, presented as (C [S, D], F, M, n), corresponding to ‘complete’, ‘single-copy’, ‘duplicated’, ‘fragmented’, ‘missing’, and ‘n’ for the dataset size, serves as a metric for genome completeness and quality. While highly useful, BUSCO scores should be interpreted contextually, as some organisms may naturally lack certain BUSCO genes due to gene losses. Beyond quality assessment of genomic data, BUSCO applications include evaluation and training of gene predictors, comparative genomics, and streamlined phylogenomics (31,32).

Here, we announce a major update to the BUSCO datasets, derived from the OrthoDB updates, aimed at enhancing accuracy and broadening taxonomic coverage. This update increased the number of datasets from 83 to 332 for Bacteria, from 16 to 29 for Archaea, and from 67 to 109 for Eukaryota. Most importantly, the representative species counts underlying these datasets have been substantially expanded to reflect broader coverage of genomic diversity. The BUSCO methodology is based on evolutionary expectations for each taxonomic clade, and sampling biases can lead to skewed evaluations. With the availability of more genomes, we can now achieve more balanced sampling by limiting each genus to a maximum of five species (for above-genus datasets) and each family to no more than 100 species. We generated a BUSCO dataset for each OrthoDB orthology level with more than ten species, ensuring each dataset has fewer than 20% duplications and a completeness score above either 90% or the clade average minus two standard deviations, whichever is lower, based on scores from previous BUSCO versions of the root datasets. We have also tightened the BUSCO marker universality threshold, requiring genes to be present as a single copy in 93% of species for datasets with over 100 species, while keeping the 90% threshold for less represented clades. This profound expansion in genomic diversity is expected to yield more accurate evaluation metrics. Note that the BUSCO approach may under-score completeness in cases of lineage-specific gene loss, while over-scoring can occur when using datasets with fewer markers, e.g. of more ancestral levels.

In 2024, we introduced a faster alternative for identifying BUSCO genes in genomic sequences with the Miniprot tool (33), which has now become the default option in BUSCO ‘genome’ mode when assessing eukaryotic genomes. While using the Miniprot option accelerates analysis, particularly of large genomes, users should be aware of potential inconsistencies, such as internal stop codons within the predicted transcripts. Underlying sequencing errors may lead proper gene predictors to false negatives, while simply mapping protein-coding sequences to genomic DNA with Miniprot bypasses this. Notably, this approach may yield higher BUSCO scores for genome assemblies from error-prone long-reads, as showcased by compleasm: a Miniprot-based, independently stripped-down version of the BUSCO software that uses the same BUSCO datasets.

BUSCO (https://busco.ezlab.org) is unique in its capability to assess both eukaryotic and prokaryotic species across various data types, including gene sets, transcriptomes, genome assemblies, and metagenomic bins. Assessing MAGs can be challenging without prior knowledge of their taxonomic origin. While BUSCO can detect and assess eukaryotic microbial genomes, these would be overlooked by the prokaryote-focused CheckM tool (34). Although CheckM remains popular in bacterial genomics, BUSCO has been shown to automatically select higher-resolution datasets for evaluations (15). The use of more markers generally leads to more reliable assessments, helping to avoid over-scoring. This update, reflecting the major increase in the coverage of genomic diversity in OrthoDB, further strengthens the foundational expectations and enhances the accuracy of BUSCO assessments.

## Data access

OrthoDB resource is publicly accessible from https://www.orthodb.org. It features a web-based graphical user interface for browsing. For bulk download we provide data files and offer programmatic access via a REST API, which returns data in JSON, FASTA, or TAB formats. Additionally, OrthoDB can be directly accessed through Python and R Bioconductor packages, facilitating integration with bioinformatics workflows. The RDF SPARQL interface of OrthoDB ensures compatibility and facilitates complex federated queries across multiple SPARQL endpoints. For example, it enables querying and linking data from OrthoDB orthologs to UniProt (21), neXtProt (35), Rhea (36) reactions or STRING (37) interactions. Users can explore SPARQL code examples at https://sparql.orthodb.org/, which features clickable links to NCBI, InterPro, and GO resources. Navigation to OrthoDB records is also possible through links from FlyBase's (38) ‘Orthologs’ section, UniProt's (21) ‘Phylogenomic databases’ section, or NCBI’s (39) ‘General gene information / Homology’ or ‘Additional links / Gene LinkOut’ sections.

OrthoLoger is freely available at https://orthologer.ezlab.org, and is also accessible as a Conda package and through BioContainers. ODB-mapper, streamlining annotation of genes from newly sequenced genomes with OrthoDB’s evolutionary and functional descriptors, is a component of OrthoLoger.

BUSCO is freely available from https://busco.ezlab.org.

## Conclusions and perspectives

This update to OrthoDB significantly enhances our global coverage of genomic diversity, which is crucial to keep pace with the rapid expansion of genomic sequence data. Despite challenges from the accelerating volume of data, which exceeds current capacities for complex analyses such as *de-novo* inference of gene orthology, our genome diversity sampling approach facilitates the integration of additional genomes with reduced effort. The ODB-mapper, a component of the OrthoLoger software, streamlines the process of annotating new genomes using the functional and evolutionary descriptors provided by OrthoDB. As the field of genomics continues to expand, so too does the demand for accurate orthology assessments. OrthoDB is committed to meeting this need by enhancing the representation of taxonomic clades and expanding our toolbox for future comparative genomic studies.

## Data Availability

OrthoDB resource is publicly available from https://www.orthodb.org, OrthoLoger is freely available from https://orthologer.ezlab.org, BUSCO is freely available from https://busco.ezlab.org.

## References

[1] Gabaldon T. , Koonin E.V. Functional and evolutionary implications of gene orthology. Nat. Rev. Genet. 2013; 14:360–366. 23552219 10.1038/nrg3456 PMC5877793

[2] Koonin E.V. Orthologs, paralogs, and evolutionary genomics. Annu. Rev. Genet. 2005; 39:309–338. 16285863 10.1146/annurev.genet.39.073003.114725

[3] Linard B. , Ebersberger I., McGlynn S.E., Glover N., Mochizuki T., Patricio M., Lecompte O., Nevers Y., Thomas P.D., Gabaldon T. et al. Ten years of collaborative progress in the quest for orthologs. Mol. Biol. Evol. 2021; 38:3033–3045. 33822172 10.1093/molbev/msab098 PMC8321534

[4] Nakaya A. , Katayama T., Itoh M., Hiranuka K., Kawashima S., Moriya Y., Okuda S., Tanaka M., Tokimatsu T., Yamanishi Y. et al. KEGG OC: a large-scale automatic construction of taxonomy-based ortholog clusters. Nucleic Acids Res. 2013; 41:D353–D357. 23193276 10.1093/nar/gks1239 PMC3531156

[5] Galperin M.Y. , Wolf Y.I., Makarova K.S., Vera Alvarez R., Landsman D., Koonin E.V. COG database update: focus on microbial diversity, model organisms, and widespread pathogens. Nucleic Acids Res. 2021; 49:D274–D281. 33167031 10.1093/nar/gkaa1018 PMC7778934

[6] Hernandez-Plaza A. , Szklarczyk D., Botas J., Cantalapiedra C.P., Giner-Lamia J., Mende D.R., Kirsch R., Rattei T., Letunic I., Jensen L.J. et al. eggNOG 6.0: enabling comparative genomics across 12 535 organisms. Nucleic Acids Res. 2023; 51:D389–D394. 36399505 10.1093/nar/gkac1022 PMC9825578

[7] Kuznetsov D. , Tegenfeldt F., Manni M., Seppey M., Berkeley M., Kriventseva E.V., Zdobnov E.M. OrthoDB v11: annotation of orthologs in the widest sampling of organismal diversity. Nucleic Acids Res. 2023; 51:D445–D451. 36350662 10.1093/nar/gkac998 PMC9825584

[8] Altenhoff A.M. , Warwick Vesztrocy A., Bernard C., Train C.M., Nicheperovich A., Prieto Banos S., Julca I., Moi D., Nevers Y., Majidian S. et al. OMA orthology in 2024: improved prokaryote coverage, ancestral and extant GO enrichment, a revamped synteny viewer and more in the OMA Ecosystem. Nucleic Acids Res. 2024; 52:D513–D521. 37962356 10.1093/nar/gkad1020 PMC10767875

[9] Mistry J. , Chuguransky S., Williams L., Qureshi M., Salazar G.A., Sonnhammer E.L.L., Tosatto S.C.E., Paladin L., Raj S., Richardson L.J. et al. Pfam: the protein families database in 2021. Nucleic Acids Res. 2021; 49:D412–D419. 33125078 10.1093/nar/gkaa913 PMC7779014

[10] Kriventseva E.V. , Rahman N., Espinosa O., Zdobnov E.M. OrthoDB: the hierarchical catalog of eukaryotic orthologs. Nucleic Acids Res. 2008; 36:D271–D275. 17947323 10.1093/nar/gkm845 PMC2238902

[11] Merkeev I.V. , Novichkov P.S., Mironov A.A. PHOG: a database of supergenomes built from proteome complements. BMC Evol. Biol. 2006; 6:52. 16792803 10.1186/1471-2148-6-52 PMC1523204

[12] van der Heijden R.T. , Snel B., van Noort V., Huynen M.A. Orthology prediction at scalable resolution by phylogenetic tree analysis. BMC Bioinf. 2007; 8:83. 10.1186/1471-2105-8-83 PMC1838432 17346331

[13] Li L. , Stoeckert C.J. Jr., Roos D.S OrthoMCL: identification of ortholog groups for eukaryotic genomes. Genome Res. 2003; 13:2178–2189. 12952885 10.1101/gr.1224503 PMC403725

[14] Emms D.M. , Kelly S. OrthoFinder: phylogenetic orthology inference for comparative genomics. Genome Biol. 2019; 20:238. 31727128 10.1186/s13059-019-1832-y PMC6857279

[15] Manni M. , Berkeley M.R., Seppey M., Simao F.A., Zdobnov E.M. BUSCO update: novel and streamlined workflows along with broader and deeper phylogenetic coverage for scoring of eukaryotic, prokaryotic, and viral genomes. Mol. Biol. Evol. 2021; 38:4647–4654. 34320186 10.1093/molbev/msab199 PMC8476166

[16] Kriventseva E.V. , Tegenfeldt F., Petty T.J., Waterhouse R.M., Simao F.A., Pozdnyakov I.A., Ioannidis P., Zdobnov E.M. OrthoDB v8: update of the hierarchical catalog of orthologs and the underlying free software. Nucleic Acids Res. 2015; 43:D250–D256. 25428351 10.1093/nar/gku1220 PMC4383991

[17] Schoch C.L. , Ciufo S., Domrachev M., Hotton C.L., Kannan S., Khovanskaya R., Leipe D., McVeigh R., O’Neill K., Robbertse B. et al. NCBI Taxonomy: a comprehensive update on curation, resources and tools. Database (Oxford). 2020; 2020:baaa062. 32761142 10.1093/database/baaa062 PMC7408187

[18] O’Leary N.A. , Wright M.W., Brister J.R., Ciufo S., Haddad D., McVeigh R., Rajput B., Robbertse B., Smith-White B., Ako-Adjei D. et al. Reference sequence (RefSeq) database at NCBI: current status, taxonomic expansion, and functional annotation. Nucleic Acids Res. 2016; 44:D733–D745. 26553804 10.1093/nar/gkv1189 PMC4702849

[19] Sayers E.W. , Cavanaugh M., Clark K., Pruitt K.D., Sherry S.T., Yankie L., Karsch-Mizrachi I. GenBank 2024 update. Nucleic Acids Res. 2024; 52:D134–D137. 37889039 10.1093/nar/gkad903 PMC10767886

[20] Harrison P.W. , Amode M.R., Austine-Orimoloye O., Azov A.G., Barba M., Barnes I., Becker A., Bennett R., Berry A., Bhai J. et al. Ensembl 2024. Nucleic Acids Res. 2024; 52:D891–D899. 37953337 10.1093/nar/gkad1049 PMC10767893

[21] UniProt Consortium UniProt: the universal protein knowledgebase in 2023. Nucleic Acids Res. 2023; 51:D523–D531. 36408920 10.1093/nar/gkac1052 PMC9825514

[22] Gene Ontology Consortium Aleksander S.A. , Balhoff J., Carbon S., Cherry J.M., Drabkin H.J., Ebert D., Feuermann M., Gaudet P., Harris N.L. et al. The gene ontology knowledgebase in 2023. Genetics. 2023; 224:iyad031. 36866529 10.1093/genetics/iyad031 PMC10158837

[23] Paysan-Lafosse T. , Blum M., Chuguransky S., Grego T., Pinto B.L., Salazar G.A., Bileschi M.L., Bork P., Bridge A., Colwell L. et al. InterPro in 2022. Nucleic Acids Res. 2023; 51:D418–D427. 36350672 10.1093/nar/gkac993 PMC9825450

[24] Kanehisa M. , Furumichi M., Sato Y., Kawashima M., Ishiguro-Watanabe M. KEGG for taxonomy-based analysis of pathways and genomes. Nucleic Acids Res. 2023; 51:D587–D592. 36300620 10.1093/nar/gkac963 PMC9825424

[25] Waterhouse R.M. , Zdobnov E.M., Kriventseva E.V. Correlating traits of gene retention, sequence divergence, duplicability and essentiality in vertebrates, arthropods, and fungi. Genome Biol. Evol. 2011; 3:75–86. 21148284 10.1093/gbe/evq083 PMC3030422

[26] Waterhouse R.M. , Tegenfeldt F., Li J., Zdobnov E.M., Kriventseva E.V. OrthoDB: a hierarchical catalog of animal, fungal and bacterial orthologs. Nucleic Acids Res. 2013; 41:D358–D365. 23180791 10.1093/nar/gks1116 PMC3531149

[27] Junier T. , Zdobnov E.M. The Newick utilities: high-throughput phylogenetic tree processing in the UNIX shell. Bioinformatics. 2010; 26:1669–1670. 20472542 10.1093/bioinformatics/btq243 PMC2887050

[28] Cosentino S. , Iwasaki W. SonicParanoid: fast, accurate and easy orthology inference. Bioinformatics. 2019; 35:149–151. 30032301 10.1093/bioinformatics/bty631 PMC6298048

[29] Hyatt D. , Chen G.L., Locascio P.F., Land M.L., Larimer F.W., Hauser L.J. Prodigal: prokaryotic gene recognition and translation initiation site identification. BMC Bioinf. 2010; 11:119. 10.1186/1471-2105-11-119 PMC2848648 20211023

[30] da Veiga Leprevost F. , Gruning B.A., Alves Aflitos S., Rost H.L., Uszkoreit J., Barsnes H., Vaudel M., Moreno P., Gatto L., Weber J. et al. BioContainers: an open-source and community-driven framework for software standardization. Bioinformatics. 2017; 33:2580–2582. 28379341 10.1093/bioinformatics/btx192 PMC5870671

[31] Waterhouse R.M. , Seppey M., Simao F.A., Manni M., Ioannidis P., Klioutchnikov G., Kriventseva E.V., Zdobnov E.M. BUSCO applications from quality assessments to gene prediction and phylogenomics. Mol. Biol. Evol. 2018; 35:543–548. 29220515 10.1093/molbev/msx319 PMC5850278

[32] Manni M. , Berkeley M.R., Seppey M., Zdobnov E.M. BUSCO: assessing genomic data quality and beyond. Curr. Protoc. 2021; 1:e323. 34936221 10.1002/cpz1.323

[33] Li H. Protein-to-genome alignment with miniprot. Bioinformatics. 2023; 39:btad014. 36648328 10.1093/bioinformatics/btad014 PMC9869432

[34] Parks D.H. , Imelfort M., Skennerton C.T., Hugenholtz P., Tyson G.W. CheckM: assessing the quality of microbial genomes recovered from isolates, single cells, and metagenomes. Genome Res. 2015; 25:1043–1055. 25977477 10.1101/gr.186072.114 PMC4484387

[35] Zahn-Zabal M. , Michel P.A., Gateau A., Nikitin F., Schaeffer M., Audot E., Gaudet P., Duek P.D., Teixeira D., Rech de Laval V. et al. The neXtProt knowledgebase in 2020: data, tools and usability improvements. Nucleic Acids Res. 2020; 48:D328–D334. 31724716 10.1093/nar/gkz995 PMC7145669

[36] Bansal P. , Morgat A., Axelsen K.B., Muthukrishnan V., Coudert E., Aimo L., Hyka-Nouspikel N., Gasteiger E., Kerhornou A., Neto T.B. et al. Rhea, the reaction knowledgebase in 2022. Nucleic Acids Res. 2022; 50:D693–D700. 34755880 10.1093/nar/gkab1016 PMC8728268

[37] Szklarczyk D. , Kirsch R., Koutrouli M., Nastou K., Mehryary F., Hachilif R., Gable A.L., Fang T., Doncheva N.T., Pyysalo S. et al. The STRING database in 2023: protein-protein association networks and functional enrichment analyses for any sequenced genome of interest. Nucleic Acids Res. 2023; 51:D638–D646. 36370105 10.1093/nar/gkac1000 PMC9825434

[38] Ozturk-Colak A. , Marygold S.J., Antonazzo G., Attrill H., Goutte-Gattat D., Jenkins V.K., Matthews B.B., Millburn G., Dos Santos G., Tabone C.J. et al. FlyBase: updates to the Drosophila genes and genomes database. Genetics. 2024; 227:iyad211. 38301657 10.1093/genetics/iyad211 PMC11075543

[39] Sayers E.W. , Beck J., Bolton E.E., Brister J.R., Chan J., Comeau D.C., Connor R., DiCuccio M., Farrell C.M., Feldgarden M. et al. Database resources of the National Center for Biotechnology Information. Nucleic Acids Res. 2024; 52:D33–D43. 37994677 10.1093/nar/gkad1044 PMC10767890

